# *In vivo* photothermal optical coherence tomography of endogenous and exogenous contrast agents in the eye

**DOI:** 10.1038/s41598-017-10050-5

**Published:** 2017-08-23

**Authors:** Maryse Lapierre-Landry, Andrew Y. Gordon, John S. Penn, Melissa C. Skala

**Affiliations:** 1 Department of Biomedical Engineering, Vanderbilt University, Nashville, TN USA; 2 Morgridge Institute for Research, Madison, WI USA; 3 Department of Molecular Physiology and Biophysics, Vanderbilt University Medical Center, Nashville, TN USA; 4 Department of Ophthalmology and Visual Sciences, Vanderbilt University Medical Center, Nashville, TN USA; 5 Department of Biomedical Engineering, University of Wisconsin, Madison, WI USA

## Abstract

Optical coherence tomography (OCT) has become a standard-of-care in retinal imaging. OCT allows non-invasive imaging of the tissue structure but lacks specificity to contrast agents that could be used for *in vivo* molecular imaging. Photothermal OCT (PT-OCT) is a functional OCT-based technique that has been developed to detect absorbers in a sample. We demonstrate *in vivo* PT-OCT in the eye for the first time on both endogenous (melanin) and exogenous (gold nanorods) absorbers. Pigmented mice and albino mice (n = 6 eyes) were used to isolate the photothermal signal from the melanin in the retina. Pigmented mice with laser-induced choroidal neovascularization lesions (n = 7 eyes) were also imaged after a systemic injection of gold nanorods to observe their passive accumulation in the retina. This experiment demonstrates the feasibility of PT-OCT to image the distribution of both endogenous and exogenous absorbers in the mouse retina.

## Introduction

Optical coherence tomography (OCT) is a standard-of-care retinal imaging technique in the clinic, and is widely used to study retinal diseases in pre-clinical research^1^. In patients, OCT can diagnose and monitor diseases such as age-related macular degeneration (AMD)^2^, diabetic retinopathy^3^ and glaucoma^4^. In animal models, OCT is used to investigate new retinal therapies and understand disease progression^5, 6^. One of the strengths of OCT is its ability to visualize individual retinal layers, which has been validated by comparing OCT images of the retina to histology for both healthy and diseased states^7–9^. Quantitative diagnostic applications for OCT have been developed based on the segmentation and measured thickness of these layers^10^. However, automated segmentation can be difficult or inaccurate when layers are severely disturbed, such as in cases of neovascularization (e.g. wet age-related macular degeneration) or detachment/displacement of the retinal pigment epithelium (e.g. drusen)^11^.

Functional information could be used not only to increase the contrast of certain layers of the retina on OCT images and facilitate segmentation, but also to provide new disease markers, and develop new drug carriers. For example, OCT angiography (OCTA)^12, 13^ is used to image blood vessels and quantify blood flow in the retina, polarization-sensitive OCT (PS-OCT)^14, 15^ provides contrast based on the birefringence of the retina, and optical coherence elastography (OCE) can be used to measure the mechanical properties of the cornea^16^. A molecular OCT technique that detects endogenous and exogenous contrast agents would provide complimentary information to structural OCT, OCTA, PS-OCT and OCE. Such a multifunctional, OCT-based approach would be beneficial for eye imaging since it is impractical to focus multiple instruments at once on the retina, and successive imaging using different instruments increases imaging time and requires image registration.

Multiple techniques have been proposed to enable OCT-based molecular imaging. OCT in its traditional form is a poor candidate for molecular imaging because of its source of contrast, scattering due to variations in index of refraction, which does not significantly change between small molecules (e.g. antibodies, most fluorescent dyes). Contrast agents can be used with OCT to increase the backscattered signal in regions of interest^17^, but the detection specificity of these contrast agents is poor against a scattering background^18^ such as a neovascular lesion in AMD models. Thus techniques such as spectroscopic^19^, pump-probe^20, 21^, magnetomotive^22^, diffusion-sensitive^23^ and photothermal OCT^24, 25^ have been developed. However, few of those methods have been applied to the eye. We thus propose photothermal OCT (PT-OCT) to image endogenous and exogenous absorbers in the eye, effectively adding new sources of contrast to structural OCT.

In this study, we used PT-OCT to image melanin and gold nanorods in the mouse eye. Sensitivity to an endogenous contrast agent is particularly useful in the eye, since few probes are FDA approved (namely indocyanine green), and contrast delivery is limited by the blood-retinal barrier. In pigmented animals, melanin is naturally present in the retinal pigment epithelium (RPE) and choroid, two layers of the retina, and its concentration and distribution can be indicative of different disease states, such as melanoma and age-related macular degeneration^26^. Increased contrast from melanin could also facilitate automatic segmentation of the RPE. Since melanin is an absorber in the near-infrared, detection with PT-OCT can be done directly, without the need for additional tags or dyes.

New exogenous contrast agents are being developed and non-invasive imaging would be particularly useful to assess their potential for *in vivo* applications. Gold nanoparticles have been investigated as possible contrast agents and drug carriers for the eye^5, 27–32^. There is a need for *in vivo* testing of those new particles to understand toxicity and biodistribution, but also because *ex situ* methods can cause particle aggregation artifacts^33^. However, current standards of quantifying biodistribution of gold nanoparticles, namely instrumental neutron activation analysis (INAA) and inductively coupled plasma mass spectrometry (ICP-MS), are labor intensive and destructive to the sample^34^. Thus, a new imaging method to non-invasively detect gold nanoparticles in the eye would bring complimentary information to the current *ex vivo* methods.

Herein, we demonstrate PT-OCT in the eye for the first time, as a non-invasive OCT-based method to detect endogenous (melanin) and exogenous (gold nanorods) contrast agents, respectively, in the retinal pigment epithelium and in choroidal neovascular lesions.

## Photothermal optical coherence tomography

**Figure 1:**
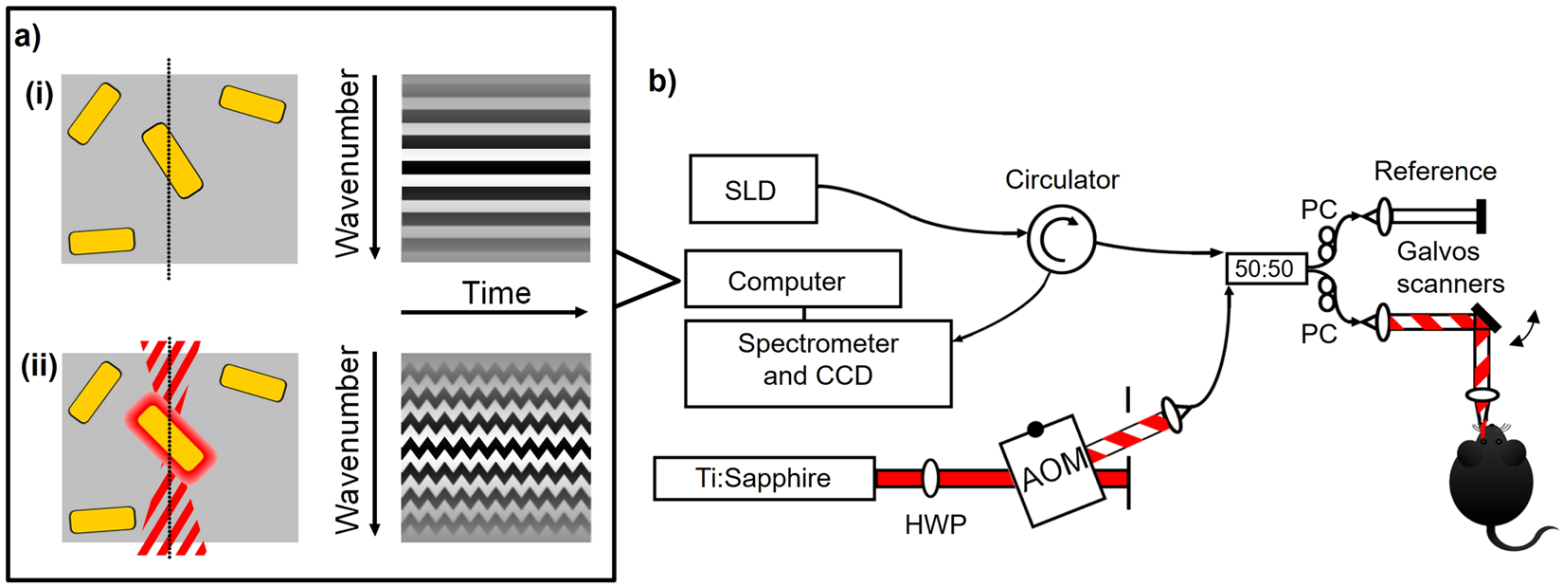
Photothermal OCT principle and instrumentation. (**a**) Illustration of the OCT raw signal over time (i) in the absence of a photothermal beam and (ii) in the presence of an amplitude-modulated photothermal beam for a sample containing some gold nanorods (yellow). (**b**) PT-OCT instrumentation. Imaging beam (SLD) centered at λ = 860 nm is divided between the reference and sample arm using a 50:50 fiber coupler. The photothermal beam (Ti:Sapphire, λ = 750 nm) is amplitude modulated with an acousto-optic modulator (AOM), and coupled to the sample arm. A circulator redirects the light from the sample to the detector. HWP: Half-wave plate. PC: polarization controller.

PT-OCT^24, 25^ adds contrast to traditional OCT images using endogenous or exogenous absorbers, such as melanin, indocyanine green or gold nanoparticles. During a PT-OCT scan, an additional laser is tuned to the absorption peak of the contrast agent or endogenous absorber, amplitude-modulated over time, and focused onto the sample. The light from the photothermal laser is then absorbed by the contrast agent or endogenous absorber and transformed into heat, which creates a local temperature variation (~1–3 °C)^35^. The temporary increase in temperature causes an elastic expansion of the sample and a local change in index of refraction. Both effects result in a change in optical path length around the absorber. Repeated OCT line scans (M-scan) are taken over time over multiple heating and cooling cycles for each sample position. OCT is sensitive to changes in optical path length^36^, as seen in the following equation showing the relationship between the change in OCT phase (ΔΦ) and the change in optical path length (ΔOPL) as a function of depth (z):

1 $${\rm{\Delta }}{\rm{\Phi }}=\frac{4\pi n{\rm{\Delta }}OPL(z)}{{\lambda }_{0}}$$

where *n* is the index of refraction of the sample and *λ*_0_ is the center wavelength of the OCT source^35, 37^. In the absence of an absorber or photothermal laser, the signal is constant over time (see Fig. 1a–i) except for small variations over time due to noise and phase decorrelation. However, if both contrast agent and amplitude-modulated beams are present, the OCT phase signal will vary as the optical path length changes^37^ (see Fig. 1a–ii). Multiple oscillations are collected (~35 oscillations take <10 ms) and the PT-OCT signal will be defined as the average oscillation amplitude acquired for one pixel. At the post-processing stage, the cyclical change in phase in the OCT signal is identified and the magnitude of the signal is quantified for each pixel. Basic PT-OCT instrumentation is composed of an additional photothermal laser that is amplitude-modulated and coupled to a standard spectral domain OCT system (see Fig. 1b). PT-OCT has been demonstrated *in vitro* ^38, 39^, *ex vivo* ^40, 41^ and *in vivo* ^37, 42^ on a multitude of contrast agents, such as gold nanospheres^25^, gold nanoshells^40^, gold nanorods^37, 42^, carbon nanotubes^39^, indocyanine green^43, 44^, melanin^35, 45^ and blood^46, 47^. The PT-OCT signal is quantitative, proportional to the photothermal laser power and to the absorber concentration, and has been characterized and modeled in previous studies^35, 37, 48^. To our knowledge PT-OCT has never been demonstrated in the eye.

## Results

### PT-OCT imaging of melanin in the retina

**Figure 2:**
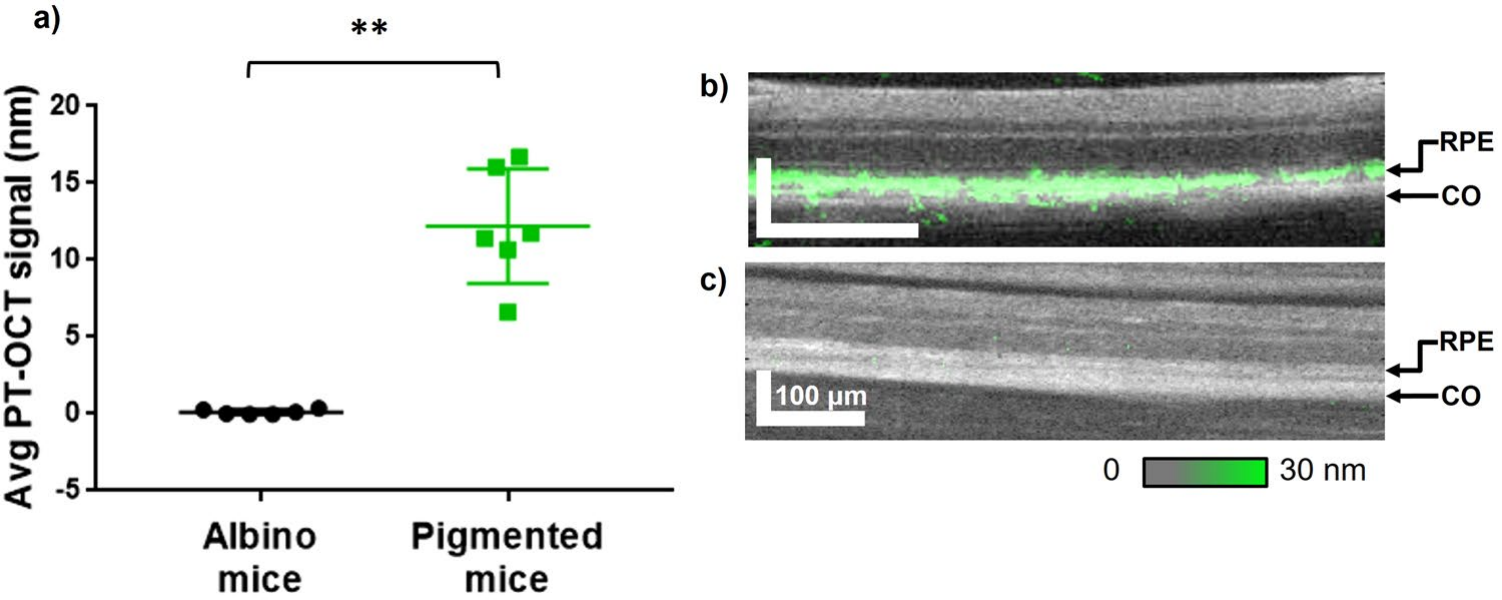
*In vivo* PT-OCT of melanin. (**a**) Average PT-OCT signal from the retina for albino mice (no melanin) and pigmented mice (melanin) cohorts (n = 6 eyes per group). Photothermal laser power at 8 mW. Mean with standard deviation shown. **p < 0.01. (**b**) Example OCT B-scan of a pigmented mouse retina (grayscale) with PT-OCT signal overlaid (green). (**c**) Example OCT B-scan of an albino mouse retina (grayscale) with PT-OCT signal overlaid (green, no signal visible). The OCT images show fewer retinal layers than in a typical OCT B-scan of a human retina because of the smaller dimensions of a mouse retina and the axial resolution of standard 860 nm OCT systems, which are also used for human retina imaging. The PT-OCT signal represents the change in optical path length, in units of nm. Scale bar: 100 μm. RPE: retinal pigment epithelium. CO: choroid.

Pigmented mice (C57BL/6, n = 6 eyes) and albino mice (BALB/c, n = 6 eyes) with unperturbed retina (no lesions) and no contrast agent injection were imaged using PT-OCT to isolate the photothermal signal from melanin. OCT and PT-OCT images were acquired while the mice were kept under anesthesia. As seen in Fig. 2a, a strong photothermal signal was detected from the RPE and choroid of the pigmented mice, while no signal above the noise floor was detected in the albino mice. The PT-OCT signal is quantified as the change in optical path length, in units of nanometers. The signal is proportional to absorber concentration, photothermal laser power, and varies between contrast agents with different absorption coefficients. In this experiment, a high signal is interpreted as a high concentration of melanin. We have found a significant difference (p < 0.01, Mann-Whitney U test) between the two experimental groups. An example B-scan for a pigmented mouse retina (Fig. 2b) and an albino mouse retina (Fig. 2c) is also shown. The location of the PT-OCT signal in depth is in accordance with the location of the RPE. This strongly indicates that melanin in the RPE can be detected with PT-OCT.

**Figure 3:**
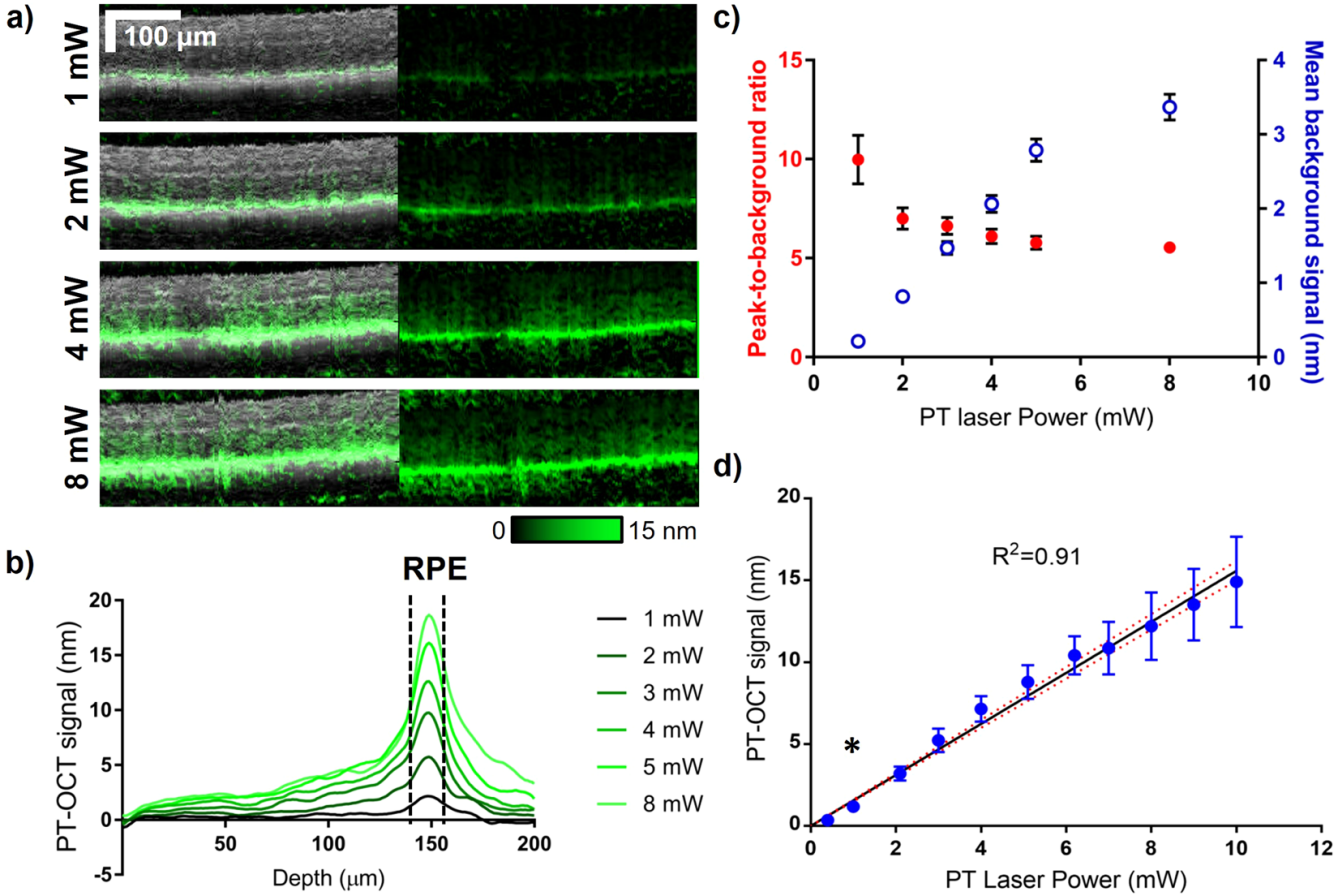
*In vivo* PT-OCT of melanin for different photothermal laser powers. (**a**) Example OCT B-scans (grayscale, left) of a pigmented mouse retina with corresponding PT-OCT signal (overlaid in green on the left, signal alone on the right) collected for different photothermal laser powers (1–8 mW). Scale bar: 100 μm. (**b**) Average PT-OCT signal for one B-scan (400 A-scans) collected at different photothermal laser powers (same eye as presented in (**a**)). The width at half-maximum of the signal peaks (dashed black lines) corresponds to the location of the RPE in depth. (**c**) Mean background signal (excludes the dashed region from (**b**)) and peak-to-background ratio for one example eye at different photothermal laser powers. Error bar: standard error. (**d**) Mean PT-OCT signal as a function of photothermal laser power for n = 6 eyes. Error bar: standard deviation. Linear fit y = 1.56x with 95%CI on the fit (red dashed). R^2^ = 0.91. *p < 0.05 between 1 mW and 0.4 mW (Mann-Whitney U test).

OCT-based imaging of melanin could have potential applications in medicine independently from studies centered on gold nanoparticles, since it could serve as an endogenous contrast agent. For this reason, we measured the average photothermal signal intensity from melanin across multiple mice at different photothermal laser powers (see Fig. 3). As seen in Fig. 3a, the intensity of the PT-OCT signal from the RPE, but also from the rest of the retina increases as the photothermal laser power increases. To better understand how the PT-OCT signal is distributed with depth in the retina, the average PT-OCT signal for one example eye can also be seen for different photothermal laser powers in Fig. 3b. The width of the peak produced by the melanin layer in the RPE does not seem to change with the photothermal laser power (p > 0.05, Mann-Whitney U test, full width at half maximum). However, the magnitude of the signal from the RPE increases as the photothermal laser power increases, as expected.

Most biological tissues have a small absorption coefficient in the near-infrared (optical window), but at high powers, heat propagating outward from the RPE combined with absorption by the tissue layers above the RPE and the choroid below (and additionally phase accumulation^35^) can create a non-negligible background signal. As seen in Fig. 3c, the mean background signal (excluding the signal from the RPE) rises as the photothermal power increases. Inversely, the peak-to-background ratio (maximum signal value inside the RPE over background) decreases as the photothermal power increases. This strongly suggests that in further studies, PT-OCT of melanin in the eye should be performed at low laser powers (preferably 1 mW or less). This would decrease the overall heating in the retina, decrease the background signal, increase the peak-to-background ratio, and this could be accomplished without degrading the effective axial resolution. Additionally, a power of 1 mW for a quickly scanned, low numerical aperture beam such as the photothermal laser is comparable in wavelength and power to routine OCT imaging in rodents^6^.

As a validation of our method, the PT-OCT signal of melanin was measured and averaged for n = 6 eyes for a wide-range of laser powers (0–10 mW). As predicted by theory^35, 37^, the increase in signal is linear with power increase (Fig. 3d; R^2^ = 0.91). No PT-OCT signal was detected with photothermal laser powers lower than 0.5 mW, including when the laser was turned off (0 mW). There was a statistically significant increase between the PT-OCT signal acquired at 0.4 mW (no signal) and the PT-OCT signal acquired at 1 mW. This was observed across multiple mice with variable amounts of melanin in their retina.

### PT-OCT imaging of gold nanorods in the retina

**Figure 4:**
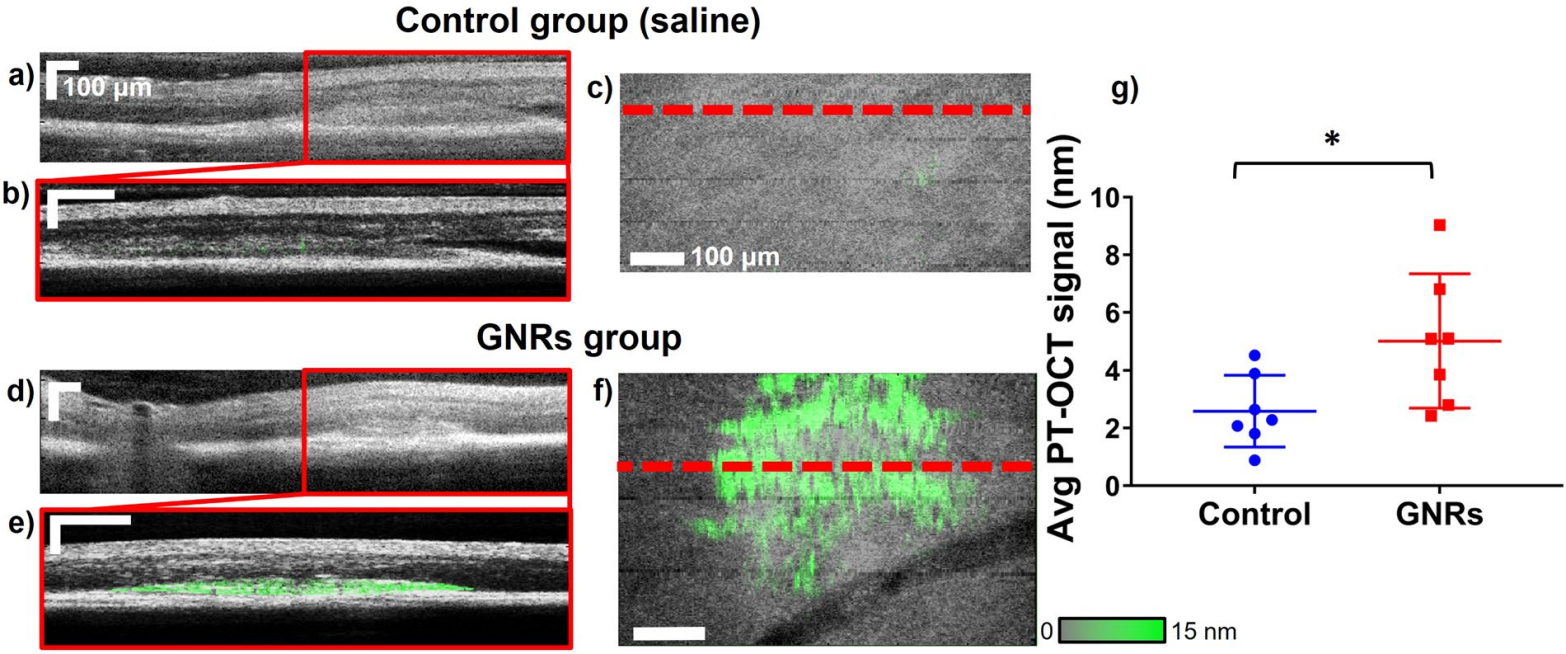
*In vivo* PT-OCT of gold nanoparticles. (**a**,**d**) OCT B-scan of the retina showing an LCNV lesion (boxed area) in, respectively, a mouse of the control group and a mouse of the gold nanorods group. (**b,e**) OCT B-scan of the LCNV lesion (grayscale) with PT-OCT signal overlaid (green) in, respectively, a mouse of the control group and a mouse of the gold nanorods group. (**c,f**) 2D *en face* mean projection of the OCT (grayscale) and PT-OCT (green) data volume in a control mouse and a mouse injected with gold nanorods. The location of the B-scan shown on the left are indicated by the red dashed line. Scale bar 100 μm. (**g**) Average PT-OCT signal normalized to the volume of the LCNV lesion for n = 7 eyes per group. Mean with standard deviation shown. *p < 0.05.

Pigmented mice (C57BL/6) underwent laser photocoagulation of the retina to create choroidal neovascular lesions. This is commonly referred to as the laser-induced choroidal neovascularization (LCNV) model, which has been widely used to study wet age-related macular degeneration^49, 50^. The lesions developed for five days after laser photocoagulation, after which fundoscopy was performed to observe the lesions. (A typical lesion five days after photocoagulation can be seen in Supplementary Fig. 1). The mice were then injected via the tail vein with either polyethylene glycol (PEG)-coated gold nanorods or saline (control group). Over time, the gold nanorods were expected to passively accumulate in the LCNV lesions, similarly to the way gold nanoparticles accumulate passively in tumors because of the enhanced permeability and retention (EPR) effect^31, 42, 51^. Six hours after injection, OCT and PT-OCT images were acquired while the mice were maintained under anesthesia. In each group, n = 7 eyes were imaged with a photothermal laser power of 8 mW. It was expected that the low concentration of the gold nanorods in the lesion would produce a smaller PT-OCT signal than the melanin in the RPE and choroid. It was thus necessary to use a higher photothermal laser power (8 mW) to reliably detect the gold nanorods at physiologically relevant concentrations. An OCT B-scan of the retina can be seen for both experimental groups in Fig. 4a and d, with the LCNV lesion identified by the red boxed area. A PT-OCT scan was taken of the boxed area, which can be seen overlaid in green on top of the corresponding OCT B-scan (grayscale) in Fig. 4b and e (the control mouse has minimal PT-OCT signal). An *en face* image of the lesion is shown in Fig. 4c and f, with the OCT signal in grayscale and the PT-OCT signal in green. The location of the B-scan shown on the previous panels is indicated by the dashed red line.

The average PT-OCT signal per pixel of the LCNV lesion for each experimental group is shown on Fig. 4g. We have found a statistically significant difference (p < 0.05, Mann-Whitney U test) between the two experimental groups. This leads us to conclude that the increase in PT-OCT signal is due to the detection of gold nanorods present in the lesion. The control group is shown to have a non-zero PT-OCT signal, which is due to melanin in the RPE. As seen in Fig. 3c (blue series) the expected mean background signal (when excluding the RPE) is approximately 3.4 nm for a photothermal laser power of 8 mW. This result matches the levels of PT-OCT signal detected in the LCNV lesions in the control group, where the RPE is also excluded (Fig. 4g, control). The gold nanorods thus significantly increase the PT-OCT signal in the LCNV lesions above the expected background signal caused by melanin at this photothermal laser power level.

## Discussion

PT-OCT is an emerging technique that adds functional contrast to traditional OCT imaging. It has been demonstrated *in vitro* ^25^, *ex vivo* ^40^ and *in vivo* ^37^ in the past on contrast agents such as gold nanoparticles^42^ and melanin^45^. However, this was the first time that PT-OCT was demonstrated in the eye, even though OCT is commonly used for retinal imaging in eye research and is a standard of care in ophthalmology^1^.

In this study, we first performed PT-OCT on cohorts of pigmented and albino mice to isolate the photothermal signal from melanin. We obtained a statistically significant increase in photothermal signal in pigmented mice, and the signal was located in depth at a retinal layer corresponding to the RPE and choroid. It was also observed that a photothermal laser power as low as 1 mW would be sufficient, and even preferable, for PT-OCT studies of melanin distribution in the retina. This is a laser power that is routinely used in animal OCT studies without damage to the retina^6^. The detection of melanin using PT-OCT could be important in the future, since it is a naturally present contrast agent in the retina and its concentration and distribution across the retina are indicative of different disease states. For example, melanin variation across the retina is observed in melanoma and age-related macular degeneration^26^. Melanin distribution could be compared to OCT intensity images and OCT angiography without the need for contrast agents or image registration. Additionally, relative concentrations of melanin across the retina could be quantified since the PT-OCT signal is directly proportional to the concentration of absorbers^37^. Even though the RPE is known to be highly back-scattering^52^, which increases the OCT signal, this change in signal is non-specific and difficult to mathematically relate to melanin concentration. PT-OCT could be an important complementary tool to understand specifically how melanin is distributed in the retina in three dimensions.

In the second part of this study, we used PT-OCT to detect the presence of gold nanorods after systemic injection of the contrast agent and passive accumulation in choroidal neovascular lesions. We detected a statistically significant increase in PT-OCT signal in the LCNV lesions in the experimental group that had been injected with gold nanorods, as compared to the control group which had levels of background signal compatible with what was detected during the melanin study. To our knowledge, this is the first time that an accumulation of gold nanorods in a neovascular lesion of the eye is detected *in vivo* using an OCT-based technology. It is usually difficult to distinguish the scattering gold nanorods against a highly scattering background, such as a lesion, from a traditional OCT intensity image^18^. However, PT-OCT can lock-in to the known frequency of the thermal oscillations created by the heated gold nanorods even when they are embedded in scattering tissue *in vivo*, as in this study.

Moving forward, *in vivo* detection of melanin in the eye using PT-OCT would allow for a dye-free, OCT-based method to detect and quantify melanin distribution in the eye. This method could be readily adopted by researchers currently studying the retina for both basic science applications, and the evaluation of new diagnostic and treatment methods. In turn, *in vivo* detection of gold nanorods in the eye using PT-OCT opens the door to molecular imaging using functionalized gold nanorods, and *in vivo* studies to characterize the biodistribution of targeted gold nanorods in the eye. For example, intravitreal injections of gold nanoparticles have been investigated for inhibition of vascular endothelial growth factor (VEGF) in the eye^5^, but no studies to our knowledge have evaluated their potential following systemic injections. PT-OCT would provide *in vivo* information about the distribution of gold nanoparticles in the eye and offer complimentary information to the current standards, INAA and ICP-MS, which both require *ex vivo* samples and are labor intensive.

In conclusion, we have demonstrated PT-OCT in the eye for the first time and have detected a signal from an endogenous contrast agent, melanin, and an exogenous contrast agent, gold nanorods, *in vivo* in mice.

## Methods

### Gold nanorod synthesis

Carboxyl-functionalized polyethylene glycol (PEG)-coated-gold nanorods with peak absorption of 750 nm (diameter: 10 nm, length: 35 nm) were purchased from Nanopartz (C12-10/750-TC-50, Loveland, USA). To achieve functionalization we utilized 1-Ethyl-3-(3-dimethylaminopropyl) carbodiimide (EDC)-mediated crosslinking of the carboxyl moiety attached to the polymer surface to a primary amine of purified rat IgG antibody^53^. Since the gold nanorods were designed to be untargeted, a purified rat IgG antibody (Thermo Fisher Scientific, Waltham, USA) with no known reactivity to murine antigens was used. This reaction occurred in the presence of N-hydroxysulfosuccinimide (sulfo-NHS) in order to increase its efficiency. Specifically 10^12^ GNRs were allowed to react for 2 hours at room temperature with EDC and s-NHS at a 10x and 25x respective concentration relative to the surface carboxyl groups in 1 mL of DI water. The solution was then centrifuged at 18,000 g for 45 minutes and the nanorods resuspended in DI water 3 times prior to the addition of a 50-fold molar overconcentration of purified rat IgG antibody. This mixture was allowed to react for 2 hours at room temperature. We then purified the functional nanorods via three additional rounds of centrifugation at 18,000 g for 45 minutes followed by resuspension in isotonic PBS prior to intravenous injection. Unreacted antibody was purified in order to assess the yield of the coupling reaction, which was approximately 20 percent.

### Animal preparation and imaging

All procedures adhered to the rules governing animal experimentation issued by Vanderbilt University Animal Care and Use and all experimental protocols were approved by the Vanderbilt Institutional Animal Care and Use Committee. Albino (BALB/c) and wild-type (C57BL/6) mice aged 8–10 weeks were used for these experiments. No randomization was used to separate wild-type mice between control and gold nanorods injected groups. The number of eyes imaged was chosen based on previous *in vivo* PT-OCT experiments^42^ and previous experiments using the LCNV model^18^. For all procedures, animals were anesthetized using isoflurane administration (2–5% in air) via nose cone. Pupils were dilated with phenylephrine (2.5%) and atropine sulfate (1%). Proparacaine (0.5%) was applied for corneal anesthesia. GenTeal lubricant eye gel (Alcon, Hünenberg, Switzerland) was applied to prevent corneal dryness. For the C57BL/6 mice used in the laser-induced choroidal neovascularization model^50^, laser photocoagulation was performed using a laser system (Carl Zeiss Meditec, Jena, Germany) with a solid-state laser (Coherent, Santa Clara, CA) at 532 nm, 100 mW power, 0.1 second pulse duration, and 100 μm spot size. Four lesions placed concentrically about the optic nerve head were created in each eye, carefully avoiding major blood vessels. Five days following lasering, white-light fundoscopy was performed using a Micron III (Phoenix Research Laboratories, Pleasanton, USA), in order to assess lesions. PT-OCT imaging was performed on the same day. Gold nanorods were administered via tail-vein injection 6 hours prior to PT-OCT imaging for the gold nanorods experimental group, and saline was administered in the same way to the control group. Each injection contained 100 μL of gold nanorods solution at 1.66 nM (100 μL injection of saline for control). PT-OCT was performed for one lesion per eye while the mouse was under anesthesia. An electric heating blanket with temperature feedback probe was used to maintain the body temperature of the animal during imaging. For the melanin detection study, all eyes imaged were included in the analysis. For the gold nanorods study, eyes with an LCNV lesion of total area >40,000 pixels were included to insure greater uniformity between replicates.

### Instrumentation and signal analysis

A spectral domain OCT system (Bioptigen Inc, 10 kHz acquisition rate) was modified for PT-OCT imaging. Using a 50:50 fiber coupler, the light from a super luminescent diode (Superlum, λ_0_ = 860 nm, 40 nm bandwidth) was combined to the light of a Titanium:Sapphire laser (Coherent, λ = 750 nm) that was amplitude-modulated (square wave, f_0_ = 500 Hz, 50% duty cycle) using a acousto-optic modulator (AOM, Brimrose). The light was then split between the reference and sample arm of the system using the 50:50 fiber coupler. A circulator (AC Photonics) was used to direct the backscattered light to a 2048 pixels CCD, and a filter was used to block λ < 800 nm light from reaching the detector. The SLD power at the sample was 2 mW and the Ti:Saph power to the sample was varied between 0–10 mW for the melanin experiment, and fixed at 8 mW for the gold nanorods experiment. A telecentric lens for mice retinal imaging (Bioptigen) was used to focus the light. The resulting system had an 8.1 μm axial resolution in air and approximately 6 μm in the retina^54^. For each PT-OCT A-scan, 700 repeated temporal OCT A-scans (M-mode imaging) were acquired. After image acquisition, the data was analyzed using MATLAB. The data was first resampled from wavelength to wavenumber, dispersion corrected^55^ and background subtracted. A Chirp-Z transform was used to convert the wavenumber dimension into spatial domain. At each depth over the 700 repeated A-scans, the first temporal derivative of the phase data was found. It was observed that 700 repeated A-scans lead to good signal sensitivity (~0.5 nm) and that a higher number of repeated A-scans did not further improve the sensitivity. A Fourier transform was used to convert the phase oscillations from the time domain to the frequency domain and the peak corresponding to the Ti:Saph modulation frequency (500 Hz) was identified. The amplitude of the peak was taken to be the PT-OCT signal intensity and the amplitude away from the peak was taken to be the noise floor. Final images were constructed by subtracting the noise floor from the signal intensity. The resulting PT-OCT signal is expressed as a change in optical path length, in units of nanometers. The optical path length variations are caused by the local changes in index of refraction and elastic expansions of the tissue due to the cyclical variations in temperature. A more detailed description of PT-OCT signal and of the data-processing algorithm is found in Tucker-Schwartz *et al*.^37^.

### Image correction protocol

To determine if PT-OCT is able to detect gold nanorods accumulating in LCNV lesions after a systemic injection, the RPE and choroid had to be excluded from our image analysis for the subsequent experiments. This was done so that variations across eyes in melanin distribution would not affect the PT-OCT signal detected in the gold nanorods experiment. Illustrations of the image analysis process can be seen in Supplementary Fig. 2.

First the OCT and PT-OCT B-scans that were acquired simultaneously are corrected for breathing artifacts (Supplementary Fig. 2a–d). An algorithm adapted from Guizar-Sicairos *et al*.^56^ is used to translate independently each OCT A-scan in depth so they align best with the geometry of the retina and the effects of breathing are removed. The algorithm uses a standard OCT volume scan acquired earlier during the experiment (fast acquisition, no oversampling over time, no breathing artifact) as a reference image. The same vertical offset used to correct an individual A-scan on the OCT image is applied to the corresponding A-scan on the PT-OCT image, effectively correcting it too. This reduces the up and down motion of the breathing artifacts but does not re-establish missing A-scans due to breathing (see Supplementary Fig. 2e). One-dimensional linear interpolation is used to correct those regions (see Supplementary Fig. 2f).

Once the breathing artifacts have been corrected, the LCNV lesion is selected manually on each OCT B-scan based on the tissue structure (Supplementary Fig. 2g). The selected regions of each PT-OCT scan (Supplementary Fig. 2h) were used for the remaining analysis. The experimental group was known to the user during manual selection (no blinding) but the selection was done based on the tissue structure shown on the OCT B-scan alone, without any PT-OCT signal shown. The PT-OCT signal was calculated only after all manual selection had been performed for all experimental groups.

### Damage to the retina

Based on the photothermal laser wavelength (750 nm), illumination time (2 ms per sample location) and power (up to 8 mW), it was determined that there was a risk of thermal damage to the retina if the temperature rose by 10 °C or more at any time during our experiment^57^. The temperature distribution was modeled^35^ based on the experimental A-scans that we have obtained at high laser powers, and it was determined that at no point during the experiment was the retina at 10 °C or more above basal temperature. The photothermal laser is pulsed (100 fs) and considered quasi-CW for the purpose of the experiment. In phantoms, we have not observed a deterioration of the photothermal signal during repeated exposition of melanin phantoms or gold nanorods phantoms to the photothermal laser (tested with powers up to 40 mW). For this reason we do not expect any significant damages to the melanin or the gold nanorods by the pulsed laser. It is possible for the pulse laser to have caused mechanical damages to the retina, but none was observed in an OCT B-scan of the mouse retina post-experiment. We are not aware of a pathway leading to photochemical damage at the cellular or molecular levels for a sample exposed to near infrared light^57^. By themselves, gold nanorods have not shown signs of toxicity to the retina^31, 58^. Thus we do not expect that permanent retinal damage was caused during the course of this experiment, but more studies would be required.

### Significance tests

Statistical significance for the increase in PT-OCT signal between pigmented and albino mice (Fig. 2) was based on the P-value (p = 0.0022) obtained from a two-tailed Mann-Whitney U test. The Mann-Whitney U test is the non-parametric equivalent to the Student’s t-test. A non-parametric test was used because of the low number of samples (n = 6 eyes from three different mice in each experimental group). Other assumptions of the Mann-Whitney U test were met. Statistical significance for the increase in PT-OCT signal between 1 mW and 0.4 mW groups (Fig. 3d) and between control and mice injected with gold nanorods (Fig. 4) was also determined using a two-tailed Mann-Whitney U test. Respective p-values of p = 0.0087 and 0.0262 were obtained. For the difference between 1 mw and 0.4 mW, n = 6 eyes from 3 different mice were used. For the comparison between gold nanorods and control groups, n = 7 eyes were used from 4 different mice in each experimental group.

### Data availability

The data that support the findings of this study are available from the corresponding author upon reasonable request.

### Code availability

The custom computer codes (MATLAB) used in this study are available from the corresponding author upon reasonable request.

## Electronic supplementary material

Supplementary Figures
