# Supplementary material for: In vivo photothermal optical coherence tomography of endogenous and exogenous contrast agents in the eye
Source: Sci Rep. 2017 Aug 23;7:9228. doi: 10.1038/s41598-017-10050-5 (PMC5569082; doi:10.1038/s41598-017-10050-5)
Supplement: Supplementary file 1 — Supplementary Figures [file 41598_2017_10050_MOESM1_ESM.pdf]

Supplementary Figures

In vivo photothermal optical coherence tomography of endogenous and exogenous contrast agents in the eye

Maryse Lapierre-Landry*[1,2], Andrew Y. Gordon[3], John S. Penn[3,4], Melissa C. Skala*[2,5]

[1] Department of Biomedical Engineering, Vanderbilt University, Nashville, TN
[2] Morgridge Institute for Research, Madison, WI
[3] Department of Molecular Physiology and Biophysics, Vanderbilt University Medical Center, Nashville, TN
[4] Department of Ophthalmology and Visual Sciences, Vanderbilt University Medical Center, Nashville, TN
[5] Department of Biomedical Engineering, University of Wisconsin, Madison, WI
*Corresponding authors

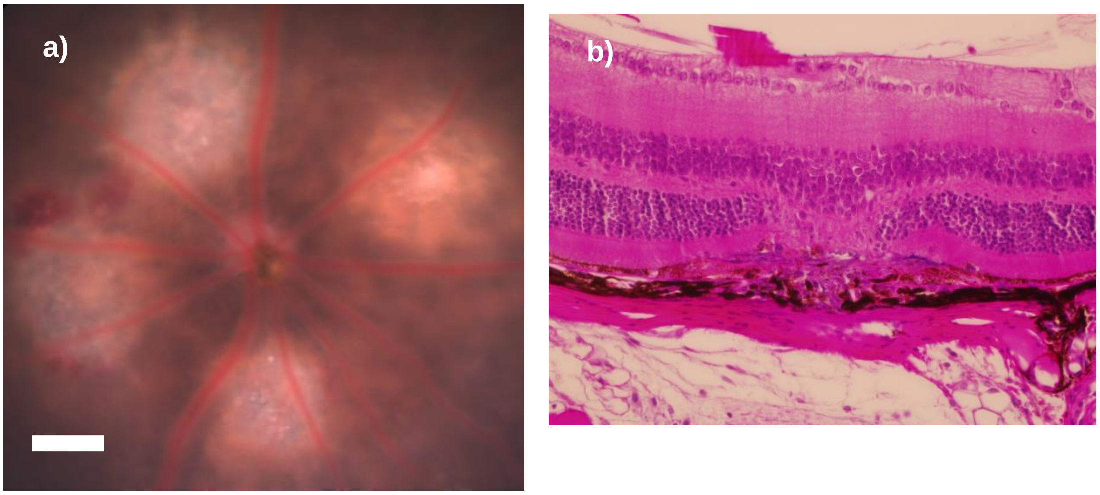

Supplementary Figure 1: Laser induced choroidal neovascularization (LCNV) lesion five days after laser photocoagulation. (a) Fundoscopy image showing four lesions around the optic nerve head. Scale bar: 400 µm. (b) Hematoxylin and eosin (H&E) stained section of an LCNV lesion. Melanin is seen in brown.

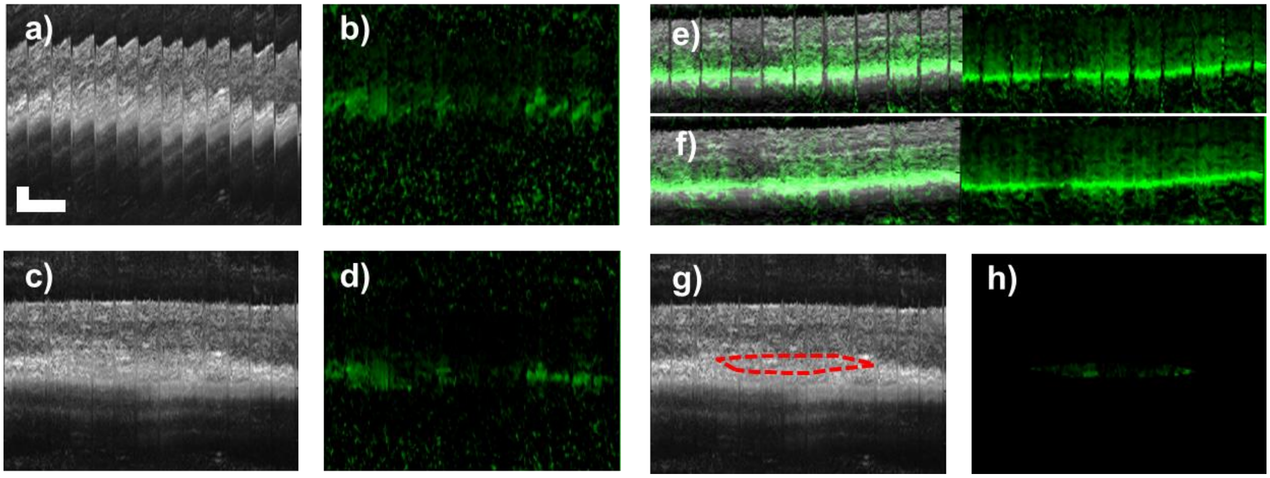

Supplementary Figure 2: Image correction protocol. (a) OCT B-scan with breathing artifacts and (b) corresponding PT-OCT B-scan. (c) OCT B-scan and (d) corresponding PT-OCT B-scan after breathing artifact correction using image registration. (e) OCT and PT-OCT B-scan with missing A-scans due to breathing artifact. (f) OCT and PT-OCT B-scan after artifact correction using one-dimensional linear interpolation. (g) Manual selection of the LCNV lesion based on the OCT corrected image. (h) Resulting masked PTOCT signal. Scale bar: 100 μm.
